# The Glasgow-Maastricht foot model, evaluation of a 26 segment kinematic model of the foot

**DOI:** 10.1186/s13047-016-0152-7

**Published:** 2016-07-08

**Authors:** Michiel Oosterwaal, Sylvain Carbes, Scott Telfer, James Woodburn, Søren Tørholm, Amir A. Al-Munajjed, Lodewijk van Rhijn, Kenneth Meijer

**Affiliations:** NUTRIM, Department of Human Movement Sciences, Maastricht University Medical Centre +, PO 5800, 6202 AZ Maastricht, The Netherlands; CAPHRI, Department of Orthopaedic Surgery, Maastricht University Medical Centre +, PO 5800, 6202 AZ Maastricht, The Netherlands; AnyBody Technology A/S, Niels Jernes Vej 10, DK-9220 Aalborg East, Denmark; The Institute for Applied Health Research, Glasgow Caledonian University, Cowcaddens Road, Glasgow, UK

**Keywords:** Kinematic foot model, Multi-segment foot model, Gait analysis

## Abstract

**Background:**

Accurately measuring of intrinsic foot kinematics using skin mounted markers is difficult, limited in part by the physical dimensions of the foot. Existing kinematic foot models solve this problem by combining multiple bones into idealized rigid segments. This study presents a novel foot model that allows the motion of the 26 bones to be individually estimated via a combination of partial joint constraints and coupling the motion of separate joints using kinematic rhythms.

**Methods:**

Segmented CT data from one healthy subject was used to create a template Glasgow-Maastricht foot model (GM-model). Following this, the template was scaled to produce subject-specific models for five additional healthy participants using a surface scan of the foot and ankle. Forty-three skin mounted markers, mainly positioned around the foot and ankle, were used to capture the stance phase of the right foot of the six healthy participants during walking. The GM-model was then applied to calculate the intrinsic foot kinematics.

**Results:**

Distinct motion patterns where found for all joints. The variability in outcome depended on the location of the joint, with reasonable results for sagittal plane motions and poor results for transverse plane motions.

**Conclusions:**

The results of the GM-model were comparable with existing literature, including bone pin studies, with respect to the range of motion, motion pattern and timing of the motion in the studied joints. This novel model is the most complete kinematic model to date. Further evaluation of the model is warranted.

## Background

The foot is comprised of 26 bones, excluding the sesamoids. Standard gait analysis considers the foot as one rigid segment connected to the shank with a ball joint [1]. Multi-segmented kinematic foot models have been developed to model intrinsic foot bone motion [2–12]. These models differ in the number of segments, ranging from two [6, 8, 11] to eight segments in the foot [5], and in the composition of these segments. The hindfoot, for example, has been modelled in at least four different ways, varying in level of detail. These include modelling the calcaneus alone [9–11], the calcaneus and talus in one segment [2, 3, 6–8], including the talus as part of the mid foot [5], or as two separate segments [12]. Furthermore, for all existing kinematic foot models the navicular bone and the three cuneiforms are modelled as a single rigid segment, an assumption that has been shown to be incorrect. For example, an in vitro study of Nester et al. [13] has shown substantial motion in three dimensions between navicular and medial cuneiform, central cuneiform and lateral cuneiform of 4.5–11.4°, 5.4–9.8° and 11.2–14.3° respectively. Current multi-segmented kinematic foot models, using standard gait analysis techniques, all use a rigid body assumption to combine individual bones in one segment, based on fixing joints that have been shown to be non-rigid.

Although the use of rigid bodies leads to the possibility to measure foot kinematics of the non-rigid joints of the chosen kinematic model, motion of joints that are modelled rigidly is neglected. When using kinematic models as an input for musculoskeletal models, these rigid bodies can be of large influence. Since a small change in muscle length can have a large influence on the strength of this muscle. Goal of this research is to generate a kinematic model that is able to capture motion in each joint, so it can be used as an input for a musculoskeletal model of the foot. No kinematic foot model has been presented to measure kinematics of all bones. Largely due to the physical dimensions of the foot bones and thus available space to attach markers, it has not been possible to capture the individual motion of all bones using standard gait analysis techniques. Current standard to capture intrinsic foot motion, uses skin mounted markers and the total number of degrees of freedom (DoF) is decreased by combining bones into segments. Another solution to decrease the number of DoF might be the use of kinematic rhythms, described by Wolf et al. [14] as functional units. These rhythms are based on functional synergies in foot motion, caused by ligamental structures overcrossing multiple joints, e.g. the transverse metatarsal ligaments couple the metatarsal motion. In general, these rhythms represent coupled motion of two or more joints.

The objective of this study was to develop a 26 segment kinematic model of the foot and ankle that used a combination of skin markers and kinematic rhythms to reduce the number of DoF. The model was tested on healthy participants and results were compared with existing data from literature.

## Methods

Detailed information on the data capturing of the measurements in this article have been published before [15]. This section gives a brief overview of the measurements performed to acquire input data for the kinematic model.

### Participants

Ten healthy participants were measured divided over two sites: Glasgow Caledonian University, UK, and Maastricht University Medical Center, the Netherlands. For both sites the local medical ethical committee approved the research. Data from four participants was not used, due to malfunction (one participant) of the measurement devices or incorrect marker capturing (three participants). Incorrect marker capturing was caused by overlapping marker tracks and missing markers that were obscured by the other leg. The demographics of the participants whose data has been used are shown in Table 1. Data from one healthy participant was used to create a ‘template’ kinematic foot model using the AnyBody Modeling System (AnyBody Technology, Aalborg, Denmark). Data from the remaining participants was analysed using subject-specific, scaled versions of the template.

**Table 1:** Anthropometrics of participants

Participant	Gender	Body mass	Foot length
GCUC01	M	76 kg	23.5 cm
GCUC02	M	74 kg	25.7 cm
GCUC03	F	85 kg	23 cm
GCUC05	M	76 kg	26.2 cm
MAS1	M	78 kg	27.5 cm
MAS2	F	58 kg	23.5 cm

GCU participants were measured at the site in Glasgow, United Kingdom, MAS participants were measured at the site in Maastricht, the Netherlands

### Data capturing

For all participants static foot surface scans (Easy Foot Scan, Orthobaltic) and motion capture data from at least three successful normal walking trials were recorded. Motion capture data included skin mounted markers (8 cameras Vicon Nexus in Maastricht, 12 cameras Qualisys in Glasgow) and ground reaction force (GRF) (Kistler 9821A SN in Maastricht, Kistler 9286B in Glasgow).

For one participant CT data of foot and ankle was acquired during an unloaded situation to provide a template foot model. The CT data was segmented using Mimics software (Materialise NV) to create 26 individual geometrical segments representing all bones of the human foot, with the exception of the sesamoid bones.

### Model implementation

#### Bones and joints

The basis for the model has been the segmented CT data. This data was used to define the geometry of the bones and position of the bones with respect to each other. The kinematic model has been implemented in the AnyBody Modeling System (AnyBody Technology, Aalborg, Denmark). The joints or kinematic links between the bones of the foot were simulated using a combination of revolute (one rotation allowed), universal (two rotations allowed) and spherical (three rotations allowed) joints, and these are detailed in Table 2. Rotation in three directions was possible for the joints in the rearfoot. For segments closer to the forefoot choices had to be made in which direction a motion was allowed, this was mainly due to the lack of space to attach markers for capturing all degrees of freedom. Previous kinematic models showed larger range of motion in the sagittal and transverse plane measurements. De Mits et al. [3] for example showed smallest range of motion in inversion/eversion for midfoot vs rearfoot and medial forefoot vs midfoot.

**Table 2:** Foot joints modelled in the Glasgow-Maastricht foot model

Joint	Type
Ankle	Revolute (from default human model in AnyBody Modeling System)
Subtalar	Revolute (from default human model in AnyBody Modeling System)
talonavicular	spherical
calcaneocuboid	spherical
1-3 cuneonavicular	universal
1-5 tarsometatarsal (TMT1-5)	universal
1-5 metatarsophalangeal (MTP1-5)	universal
1-5 interphalangeal (IP1-5)	revolute

#### Rhythms

Due to the size of the foot not all of the remaining DoF of every segment could be tracked separately. Previous studies have used the rigid body assumption to reduce the number of DoF, in the present model functional units or rhythms were used to constrain the number of DoF in the model and to allow the kinematics of all bones to be estimated with a reduced marker set. The rhythms, used in this version of the model, are given in Table 3, with additional details given in the Appendix.

**Table 3:** Rhytms

Name of the rythm	Coupled joints
IP plantar flexion	For each phalanx inter phalangeal joints
MTP plantar flexion1-5	Flexion of all MTP joints is coupled
Metatarsal tranverse arch 1-5	An arch is constructed, that couples the height of the metatarsal heads
Tarsal tranverse arch 1-5	Transverse arch coupling motion of all tarsals
Longitudinal medial arch	Coupling plantar flexion of joints in the first ray
Longitudinal lateral arch	Coupling plantar flexion of Calcaneocuboid joint and TMT5

Rythms used and short description of the rythm. A full description can be found in the Appendix

#### Inter-tarsal contact

Between the cuboidnavicular, cuneocuboid and the two intercuneiforms joints no idealised joints were defined. However, these joints do exist and a definition of the interaction between the bones within these joints is needed, to avoid the possibility for the algorithm to move bones within each other. These joints were modelled as gliding joints using an ellipsoid fitted to the lateral side of the tarsal bones. Interaction between these ellipsoids was modelled with a contact algorithm, which did not allow the ellipsoids to intersect.

#### Scaling

**Fig. 1:**
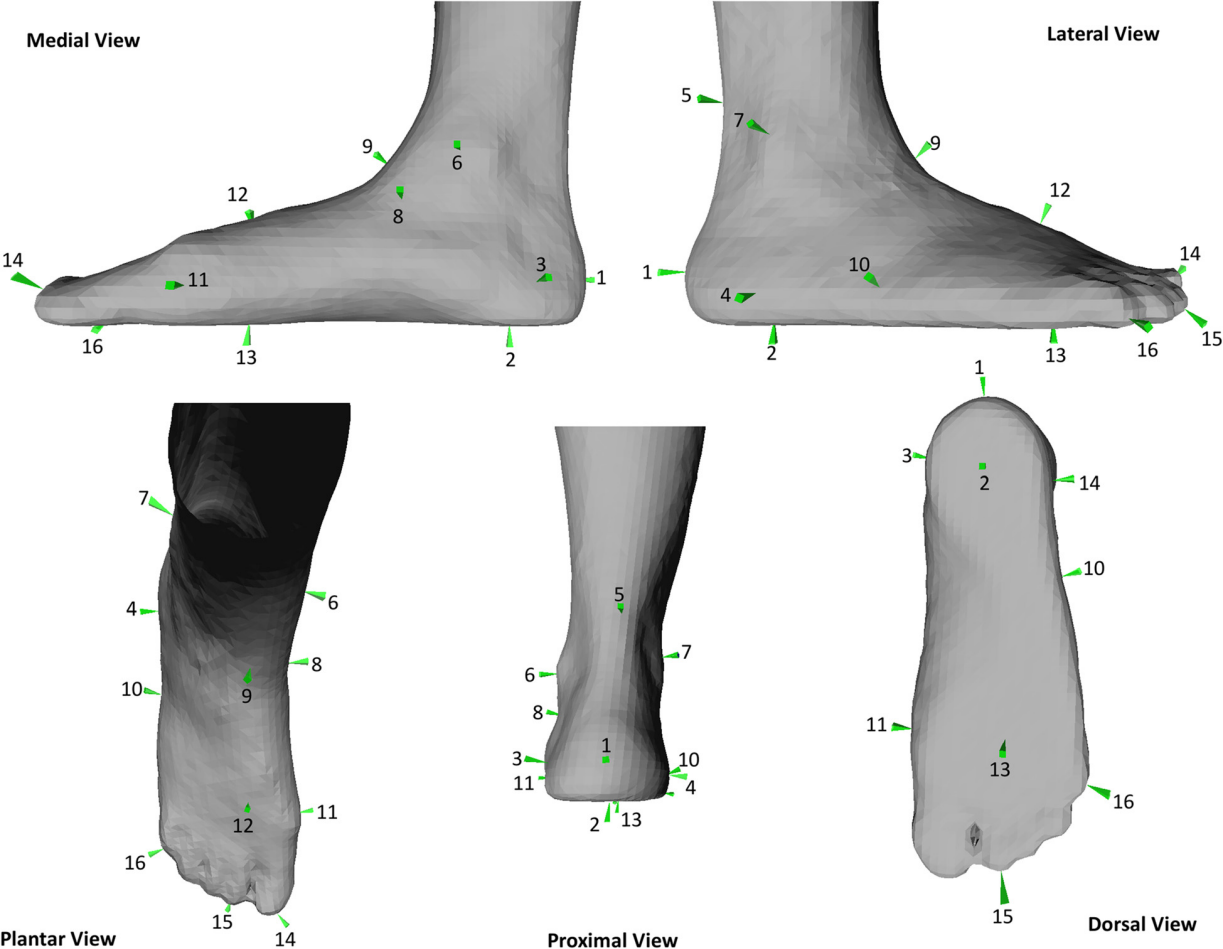
Points on which the scaling algorithm is based. *1*) Posterior Calcaneus, distal of achillis tendon *2*) Central plantar heel *3*) Heel medial *4*) Heel lateral *5*) Achilles tendon between malleoli *6*) Malleolus medial *7*) Malleolus lateral *8*) Navicular tuberosity *9*) Navicular dorsal *10*) Fifth metatarsal basis *11*) First metatarsal head, medial *12*) TMT2 dorsal *13*) MTP3 plantar *14*) Hallux tip *15*) Second toe tip *16*) Fifth toe tip

The model was scaled to each subject using the built in radial basis function (RBF) with a thin plate spline [16]. A set of 16 landmarks (see Fig. 1) on the surface scan geometry of the participant used for the template was defined, and a corresponding set was defined on each new participant’s scan. The system subsequently calculated the RBF function so that the source landmarks matched the target ones. Every point in the volume of the landmarks was scaled with this non-affine transformation.

#### Motion and simulation

The model was driven using motion capture data from all participants, three trials for each subject. A previously described marker protocol with 43 markers on the lower extremities was used [15]. The marker data was used to calculate joint angles in a kinematic analysis of an over-determinate system [17].

#### Data analysis

To compare inter-subject joint angles and to avoid dependency to anatomical neutral position, all angles were subtracted by the angle on heel strike, so at heel strike all angles were set to 0° range of motion (RoM). Data was resampled to intervals of 0.5 % of stance phase, to obtain an inter subject comparison. The mean RoM was calculated per subject over three successful trials. Minimal and maximal RoM over participants was calculated as well as minimum and maximum joint angles.

To quantify the variance in joint angles over the different participants, an adapted version of the coefficient of variation was used. Since joint angles vary around zero, the coefficient of variation would increase significantly for smaller values. Therefore the standard deviation per time step was divided by the full RoM of the corresponding joint (instead of the mean, as undertaken for the coefficient of variation). Subsequently the percentage of the stance phase was calculated, for which this adapted measure was smaller than 0.25. This measure implies the percentage of stance phase for which 68 % of the participants had a joint angle that differs less than 25 % of the range of motion of the joint. The value of 0.25 was chosen by performing a trial and error sensitivity analysis; it had to be able to distinguish joints with a small error band and those with a large error band. This measure of the variation was called COV25 (range 0–100, where 0 is a high variance and 100 a low variance).

Since the motion of some DoF are coupled in this model, only selected movements are presented. TMT1 and navicular with medial cuneiform are not shown, as they are coupled with talus navicular (plantar flexion and adduction). Distal interphalangeal joints (2–5) were not shown, since these joints are directly coupled with corresponding proximal interphalangeal joint. All other coupled DoF, as described in the Appendix are shown since these joints did not have a one-to-one relation with another joint.

## Results

**Fig. 2:**
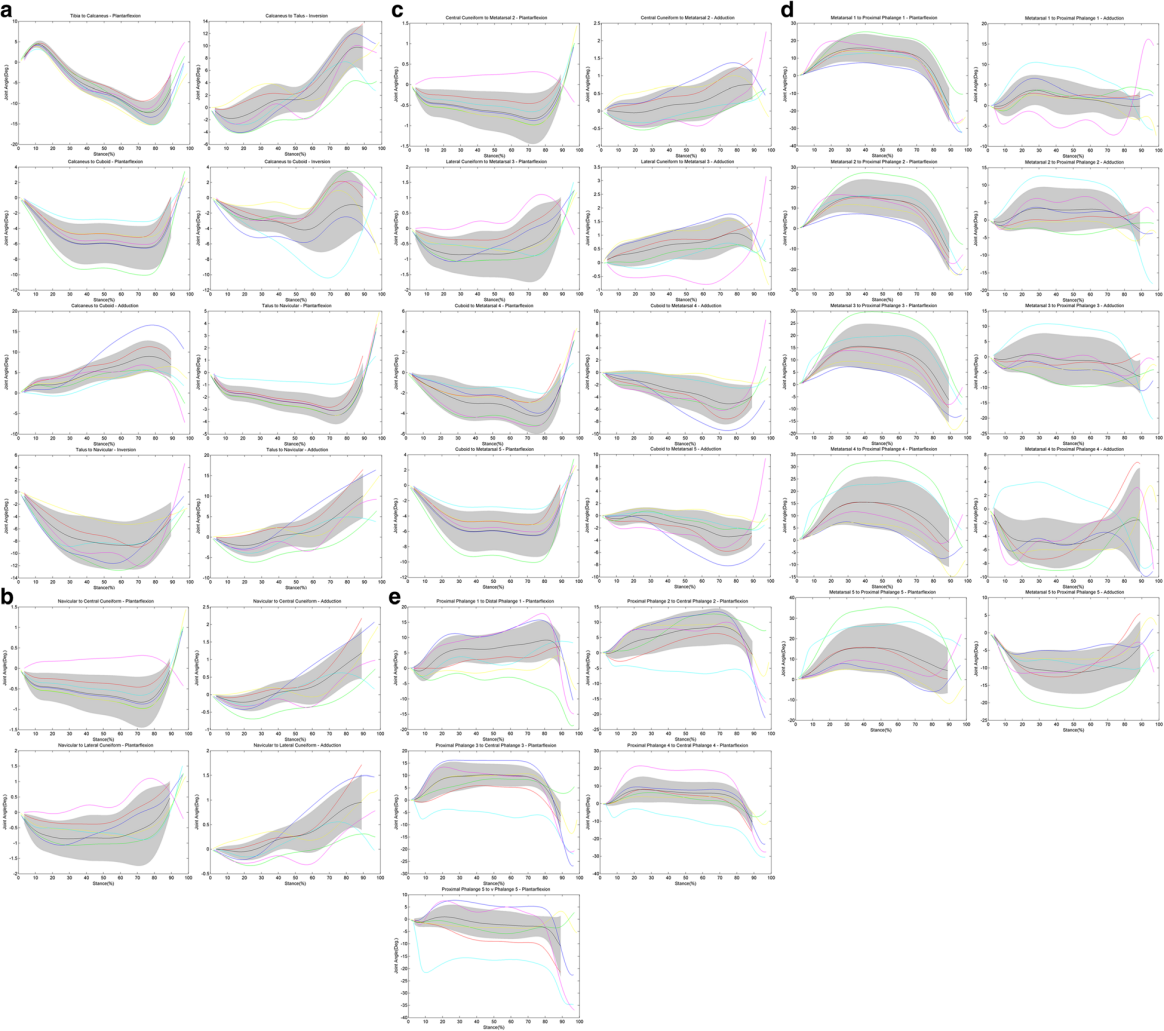
RoM for all joints. The *bold black line* is population average, *grey band* was the population standard deviation (which were only calculated when data for all participants was present on that particular time step). *Coloured lines* are averaged joint patterns per individual, where joints are grouped by: **a**) rearfoot to midfoot **b**) navicular to cuneiforms **c**) TMT **d**) MTP **e**) inter-phalangeal joints

Combined motion curves for all studied joints are presented in Fig. 2a-e. In Fig. 2a the calcaneocuboid and talonavicular joint, the two joints of Chopart’s joint, show different motions in all three directions. This difference can be seen in size and shape of the pattern. In the frontal plane, the calcaneocuboid joint shows both eversion (up to 65 % stance phase) and inversion (from 65 % stance phase), while talonavicular joint shows only eversion. In sagittal plane largest motion in talonavicular joint is in the late stance phase, while calcaneocuboid joint shows largest motion during mid stance. The largest difference is in the transverse plane, mean RoM of 9.8° (calcaneocuboid) and 14.6° (talonavicular), as can be found in Table 4.

**Table 4:** Summary of the results

	Mean RoM	Max RoM	Min RoM	Max Joint Angle	Min Joint Angle	COV25 (%)
Tibia to Talus – Plantarflexion	17.0	19.5	14.9	5.9	−16.1	100
Calcaneus to Talus – Inversion	12.9	16.2	9.3	15.1	−4.0	97
Calcaneus to Cuboid – Plantarflexion	8.3	13.1	5.0	3.3	−11.7	29
Calcaneus to Cuboid – Inversion	7.6	12.5	5.6	4.0	−11.0	56
Calcaneus to Cuboid – Adduction	9.8	15.3	6.1	15.3	−4.0	27
Talus to Navicular – Plantarflexion	6.3	8.4	2.6	5.0	−4.3	100
Talus to Navicular – Inversion	9.3	13.7	4.0	0.9	−13.7	14
Talus to Navicular – Adduction	14.6	18.6	9.6	17.8	−6.0	70
Navicular to Central Cuneiform - Plantarflexion	2.0	3.5	0.6	2.0	−1.4	85
Navicular to Central Cuneiform – Adduction	1.8	2.4	1.1	2.3	−0.7	73
Navicular to Lateral Cuneiform - Plantarflexion	2.8	7.0	1.2	4.5	−2.5	34
Navicular to Lateral Cuneiform – Adduction	1.3	1.8	0.7	1.8	−0.2	58
Central Cuneiform to Metatarsal 2 - Plantarflexion	2.0	3.5	0.6	2.0	−1.4	85
Central Cuneiform to Metatarsal 2 - Adduction	1.6	2.3	1.1	2.1	−0.6	5
Lateral Cuneiform to Metatarsal 3 - Plantarflexion	2.8	7.0	1.2	4.5	−2.5	34
Lateral Cuneiform to Metatarsal 3 - Adduction	2.1	3.0	1.0	2.6	−0.9	26
Cuboid to Metatarsal 4 – Plantarflexion	6.9	9.7	3.9	4.4	−6.2	100
Cuboid to Metatarsal 4 – Adduction	6.4	11.1	1.8	4.9	−8.2	17
Cuboid to Metatarsal 5 – Plantarflexion	8.3	13.1	5.0	3.3	−11.7	29
Cuboid to Metatarsal 5 – Adduction	5.5	10.8	2.2	6.0	−7.5	25
Metatarsal 1 to Proximal Phalange 1 - Plantarflexion	40.0	46.4	32.3	28.9	−33.7	100
Metatarsal 1 to Proximal Phalange 1 - Adduction	11.5	19.7	5.7	12.0	−10.0	12
Metatarsal 2 to Proximal Phalange 2 - Plantarflexion	33.4	40.5	27.4	31.0	−28.3	42
Metatarsal 2 to Proximal Phalange 2 - Adduction	11.5	33.7	4.9	13.3	−20.4	6
Metatarsal 3 to Proximal Phalange 3 - Plantarflexion	27.5	36.3	21.3	33.3	−22.9	29
Metatarsal 3 to Proximal Phalange 3 - Adduction	14.3	42.3	5.9	14.3	−28.1	4
Metatarsal 4 to Proximal Phalange 4 - Plantarflexion	23.4	35.8	14.1	35.8	−17.5	21
Metatarsal 4 to Proximal Phalange 4 - Adduction	11.0	16.1	9.1	8.7	−10.8	4
Metatarsal 5 to Proximal Phalange 5 - Plantarflexion	22.4	38.7	12.8	38.7	−12.8	13
Metatarsal 5 to Proximal Phalange 5 - Adduction	14.5	22.3	7.8	6.7	−22.3	19
Proximal Phalange 1 to Distal Phalange 1 - Plantarflexion	19.8	31.4	6.5	19.2	−16.4	65
Proximal Phalange 2 to Central Phalange 2 – Plantarflexion	16.1	30.4	5.9	17.6	−13.7	13
Proximal Phalange 3 to Central Phalange 3 – Plantarflexion	22.0	37.8	9.8	19.1	−19.0	70
Proximal Phalange 4 to Central Phalange 4 – Plantarflexion	25.5	48.1	16.3	21.1	−27.0	27
Proximal Phalange 5 to v Phalange 5 - Plantarflexion	22.4	42.9	8.0	17.4	−35.8	13

All data was calculated for the complete population

The navicular to cuneiform joints in Fig. 2b show small RoM of maximal 2.8° in all directions; the lateral and central cuneiform show a similar mean pattern as the talonavicular joint. However, when looking at individual participants different patterns are found in sagittal plane. In the central cuneonavicular joint, five out of six participants show a similar pattern, first dorsiflexion and in late stance phase a quick plantar flexion. While in the lateral cuneonavicular joint no consistency is found in the motion pattern of the different participants.

In the Lisfranc joint in Fig. 2c an increasing RoM is found from medial to lateral joints in sagittal plane. Motion in TMT1-3 is showing a different pattern than TMT4-5 in both directions. In the transverse plane an increasing abduction is seen in TMT 4–5, while TMT 2–3 are showing an increasing adduction. Mean RoM in sagittal plane of the GM-model for the individual MTP joints ranges from 22.4 to 33.5°, with increasing value from MTP 5 till 2 as can be seen in Fig. 2d.

In general the largest COV25 within a joint is found in sagittal plane. In the sagittal plane the largest motion is found in MTP1 (RoM = 40.0°, varying from 32.3 to 46.4°).

## Discussion

A novel kinematic foot and ankle model is presented. This model is scalable via 3D surface scanning of the foot shape, and kinematically driven by a set of 43 skin-mounted markers. To calculate kinematics for all joints connecting the 26 bones in the foot, seven rhythms are implemented. These rhythms are mathematical formulations that couple the motion of multiple bones, and are adaptable to model specific foot deformities in the future. To our knowledge this is the first study that reported on mobility of the majority of joints in the foot using skin based markers. Calculated RoM of the joints are in line with previous findings [2, 3, 5, 9, 13, 18, 19], however for some joints no comparable data is available.

### Comparison with existing literature

Full RoM of MTP1 in sagittal plane of 40.0° is comparable with results on cadaver feet [13] and existing multi-segment foot models [2, 3, 5, 18], also the timing of plantar flexion and dorsal flexion are visually comparable with existing data. Full RoM in the transverse plane of MTP1 is comparable to results from cadaver experiments [13]. However, the timing in the cadaver experiments is different, for instance in the GM-model no abduction was seen at the end of stance phase. This difference in timing might be caused by the timing of the external forces driving the cadaver experiments. Comparing with other multi-segment foot models that include MTP1 no consistency has been found between the various multi-segment foot models [2, 3, 5, 18]. As in other models, RoM for MTP1 in transverse plane in the GM-model also shows a low COV25, the reason for this could be a large inter subject variation or a DoF that is hard to measure.

Typically, the RoM of MTP joints 2–5 are not included separately in multi-segment foot models. However, MacWilliams et al. [5] used a model that separated the phalanges in medial (second and third) and lateral (fourth and fifth) phalanges. Compared to our data, MacWilliams et al. [5] showed larger RoM for the lateral phalanges in the sagittal plane. However, the shape of the curves was similar to our findings. This could be caused by the differences between the participant groups, the current study involved adults, while MacWilliams et al. were measuring on adolescents (12.49 ± 2.6 years), Nigg et al. showed a decreasing flexibility with age in the foot [20]. For the transverse plane our results are comparable with the results of MacWilliams et al. [5] for shape and magnitude. Although the GM-model shows a large inter-subject variation for MTP2-5, comparison with 1 other experiment [5] shows a moderate comparison.

Motion for individual TMT-joints has not been reported using skin mounted markers. However, MacWilliams et al. [5] did report motion between medial metatarsals and tarsal bones (mean RoM in sagittal plane 15°, in transverse plane 7°) and lateral metatarsals and tarsal bones (mean sagittal RoM 10°, in transverse plane 4°). Results of the GM-model showed a much lower RoM in sagittal plane for TMT I-V. However, transverse plane motion for TMT IV (8°) and V (7°) is higher in the GM-model compared to the MacWilliams model. The Ghent Foot Model [3] divided the TMT motion in TMT I and a combined motion for TMT II-TMT V. In this model, TMT I motion is defined with respect to all tarsal bones in contrast to the GM-model in which it is only linked to the medial cuneiform, however motion pattern and magnitude in transverse and sagittal plane differs compared to our results. In the sagittal plane an opposite motion is noticed for TMT I and TMT II – TMT V in the Ghent Foot Model, this large difference seems to be an effect of the rigid body assumption to combine Metatarsal 2–5 and all tarsals. Alternatively, Leardini et al. [9] measured combined tarsal and metatarsal bones, transverse and sagittal plane motion was in the same range as TMT IV and TMT V of the GM-model. However, the pattern is not comparable, this could be caused by the combination of all tarsals and all metatarsals into two segments by Leardini et al. [9]. Since the GM-model shows different patterns for TMTI-TMTV, the summation of these different patterns into one motion of one joint that describes all these separate motions leads to a motion that cannot be compared.

Previous kinematic foot models have taken all tarsal bones as one segment, therefore the midfoot kinematics of the GM model can only be compared with cadaver [13] and bone-pin studies [19]. Compared to cadaver studies, the RoM of medial cuneonavicular joint in the sagittal plane was lower in the GM-model during start of stance phase but was similar at the end of the stance phase. No consistent pattern was observed for this joint in the bone-pin study of Lundgren et al. [19]. In the transverse plane the cadaver and bone-pin studies measured opposite motion. Our results are closer to the bone-pin study [19]. The RoM of the other cuneonavicular joints (central and lateral cuneonavicular joint) has only been measured in cadaver experiments, for both joints sagittal joint motion was lower in the GM-model, however standard deviation in the cadaver experiments was very high (5.1° ± 9.8° and 3.6 ± 14.3°), where the consistency for the GM-model was moderate to high (0.85 and 0.34). The RoM in the transverse plane of central cuneonavicular joints was of the same magnitude and has a similar pattern of motion for the GM-model and the cadaver experiments; however the cadaver experiment showed a large variance (2.2° ± 5.4°).

The calculated RoM of talonavicular joint of the GM-model is partly comparable with previous bone pin data [19], with a late stance plantar flexion, mid stance eversion. However, transverse plane motion of the GM-model shows a different pattern, comparable to the cadaver experiments [13]. Calcaneocuboid joint motion in frontal and sagittal plane followed the same pattern as the cadaver experiments. However transverse plane motion was of a different pattern in the cadaver experiment and bone pin studies. The RoM of calcaneocuboid joint in GM-model was similar in pattern and magnitude as reported by the MacWilliams model [5], which is currently the only kinematic model that measures this joint. Other multi-segment foot models only measured complete Transverse tarsal (or Chopart’s) joint. We did not combine the calcanocuboid and the talonavicular joint RoM of the GM-model into a combined joint, since these two separate joints show a different pattern.

To the authors’ knowledge dynamic RoM of inter-phalangeal joints was not measured before, this might be because of the high resolution needed to accurately measure the motion of small bones.

All in all, no consensus was found in existing literature for most of the joints. However, the novel GM-model showed RoM’s within the variations reported across existing literature. The source of the difference within existing literature and when compared with the GM-model can be in different segment and joint definition and marker placement. Furthermore, there could be compensation in directions in which no motion was allowed, most joints have 2 DoF and no motion is allowed for eversion and inversion, while cadaver and bone-pin studies have shown physical motion in all three planes. Therefore physical motion in a non-modelled direction, can be seen by the model as a motion in a direction that is modelled, this leads to a wrong prediction of the motion.

Another source of differences between existing literature and the results of the GM-model is the different angle definition. The GM-model used joint angles in the local orientation of that joint. While cadaver experiments and bone pin studies do not model joints, but consider absolute differences between the orientations of two bones with respect to the axis of the measurement system. Therefore more distal joints will have a larger deviation, since their orientation is influenced by proximal joints.

### Limitations

Limitations of the study are: 1) the small number of participants, 2) the comparison with literature, rather than with subject specific golden standard data, 3) no separate analysis on the effect of scaling and 4) the lack of an inter- and intra-subject variations analysis.

One of the limitations of the current study is that data from six out of ten participants was used. The exclusion of three of the participants was caused by an incorrect marker tracking. This is probably caused by the number of cameras used in the experiment, since these three participants were measured at the Maastricht site, with an eight camera-set up. Due to this small number of cameras it is difficult to position the cameras. On the one hand they should be close enough to capture the small markers on the foot. On the other hand the cameras should have sufficient distance to the foot to avoid reflection of the foot and to increase the field of view. The bandwidth of this positioning is small and is a source error, which can be reduced by increasing the number of cameras or the resolution of the cameras.

The measurement of foot and ankle kinematics currently does not have a gold standard to measure bone motion in a non-invasive, three dimensional, dynamic situation. We have compared results of the GM-model with available data sources. However, a better validation would be possible when novel techniques are further developed, e.g. dynamic MRI and 3D fluoroscopy [21].

Another limitation is that no experiments have been performed to the effect of the scaling. To decrease the error caused by marker misplacement, it has been chosen to use an extra surface scan to scale the model. However, this scaling is based on 16 points on the foot and ankle, in contrast to the 30 markers used to trace the dynamic system. A sensitivity analysis should be performed to calculate the effect of the scaling.

Before using this model for clinical research it is necessary to study intra- and inter-session variations [22]. This study only focussed on the development of the model and the validation of its output. Intra session variations are expected to be small, due to the use of the kinematic analysis of an over-determinate system [17].

The rhythms coupled the motion of various joints; therefore the total number of DoF is decreased. Nevertheless, this model shows the ability of driving all segments. Due to the set-up of the model, it is possible to adapt the model to the needs of a specific research question. It is also possible to model the biomechanical aspects of a specific pathology by adapting the parameters of the rhythms or change or remove and add certain rhythms if, for example, a joint is fixed by an arthrodesis. Since the position of all bones can be computed with this kinematic model, this model will be included in a novel musculoskeletal 26 segment foot model. Since muscles are attached to all bones of the foot, motion within the foot leads to different work lines for muscles and therefore different force characteristics.

## Conclusions

In conclusion, we have developed a 26 segments kinematic foot model, which uses coupling of DoF to reduce the total number of DoF. The GM-model has showed a large inter subject variance in the kinematic results, which is in accordance to reported variances in previously reported kinematic foot models. Also in comparison with highly invasive, in-vivo, measurements, the novel model gives similar results. An application of this model is the possibility to test the effect of assumptions made in earlier models, by comparing the results of the model with current settings to a model with locked joints to simulate multi-bone segments. Another application of this model is the use as an input for a musculoskeletal foot model, giving further insights in the intrinsic muscle activation during gait.

## Appendix

### Rhythms

#### Inter phalangeal plantar flexion

A toe flexion rhythm was introduced in order to link the flexion of all toes. The kinematic measures of proximomedialphalange flexion (PIP joint flexion: φ) and distalphalange flexion (DIP joint flexion: ω) are linked together by one coefficient, so that it becomes one-DoF. For phalange 2–5, the rhythm equation can subsequently be written (in degrees):

1 $$ \begin{array}{cc}\hfill 2.2\varphi \hfill & \hfill =\omega \hfill \end{array} $$

In which the factor 2.2 is an estimation based on visual observations on healthy volunteers.

#### Metatarsophalangeal plantar flexion1-5

The five metatarsophalangeal joint flexions are linked together by three linear equations so that when the first and fifth metatarsophalangeal flexions are defined, the second, third and fourth are evenly distributed in between. The rhythm equations can subsequently be written (in degrees):

2 $$ {\theta}_2={\theta}_1\hbox{--} 1/4\left({\theta}_1\hbox{--} {\theta}_5\right) $$

3 $$ {\theta}_3={\theta}_1\hbox{--} 2/4\left({\theta}_1\hbox{--} {\theta}_5\right) $$

4 $$ {\theta}_4={\theta}_1\hbox{--} 3/4\left({\theta}_1\hbox{--} {\theta}_5\right) $$

#### Metatarsal tranverse arch 1-5

A rhythm has been implemented to control the curvature of the metatarsal transverse arch, and thus its rising and flattening. The height of the second (α), third (β) and fourth (γ) metatarsal head is measured from a base line connecting the first and fifth metatarsal head. Then the heights of the second and fourth metatarsal head are linked by a coefficient to the height of the third (Eqs. 5 and 6).

5 $$ \alpha =0.7\beta $$

6 $$ \gamma =0.9\beta $$

#### Tarsal tranverse arch 1-5

The tarsal transverse arch consisting of the three cuneiform, the cuboid, and the base of the five metatarsals is modeled to flatten like the metatarsal transverse arch during the stance phase of gait. The base line connects the plantar base of the first metatarsal and the plantar base of the fifth metatarsal. The curvature is controlled by the height of the plantar base of the second metatarsal.

#### Longitudinal medial arch

The plantar flexion angle of the talonavicular joint, naviculomedialcuneiform joint and first tarsometatarsal joint are linked by two coefficients to form a single degree of freedom. The curvature is thus controlled by the talonavicular flexion angle.

7 $$ \mathrm{TaloNavicularFlexion}=2*\mathrm{NaviculoMedCunFlexion}=2*\mathrm{T}\mathrm{M}\mathrm{T}1\mathrm{Flexion} $$

#### Longitudinal lateral arch

The longitudinal lateral arch is defined similarly by linking the flexion angle of the calcaneocuboid joint and fifth tarsometatarsal joint. The curvature is thus controlled by the calcaneocuboid plantarflexion angle.

8 $$ \mathrm{CalcaneoCuboidFlexion}=2*\mathrm{T}\mathrm{M}\mathrm{T}5\mathrm{Flexion} $$
